# Functional Analysis of Two Flavanone-3-Hydroxylase Genes from *Camellia sinensis*: A Critical Role in Flavonoid Accumulation

**DOI:** 10.3390/genes8110300

**Published:** 2017-10-31

**Authors:** Yahui Han, Keyi Huang, Yajun Liu, Tianming Jiao, Guoliang Ma, Yumei Qian, Peiqiang Wang, Xinlong Dai, Liping Gao, Tao Xia

**Affiliations:** 1 State Key Laboratory of Tea Plant Biology and Utilization, Anhui Agricultural University, Hefei 230036, Anhui, China; hyahui@163.com (Y.H.); 18326639741@163.com (T.J.); mgl1201@126.com (G.M.); qianym306@126.com (Y.Q.); wpqtea@163.com (P.W.); xinlongdai@163.com (X.D.); 2 School of Life Science, Anhui Agricultural University, Hefei 230036, Anhui, China; huluobuhashiqi@163.com (K.H.); liuyajun1228@163.com (Y.L.)

**Keywords:** *Camellia sinensis*, flavanone-3-hydroxylase, gene function, flavonoids

## Abstract

Flavonoids are major secondary metabolites in *Camellia sinensis*. Flavanone-3-hydroxylase (F3H) is a key enzyme in flavonoid biosynthesis in plants. However, its role in the flavonoid metabolism in *C. sinensis* has not been well studied. In this study, we cloned two *F3H*s from *C. sinensis*, named *CsF3Ha* and *CsF3Hb*, where *CsF3Ha* containing 1107 bases encoded 368 amino acids, and *CsF3Hb* containing 1071 bases encoded 357 amino acids. Enzymatic activity analysis showed both recombinant CsF3H enzymes in *Escherichia coli* could convert naringenin and eriodictyol into dihydrokaempferol (DHK) and dihydroquercetin (DHQ), respectively. The expression profiles showed that *CsF3Ha* and *CsF3Hb* were highly expressed in the tender leaves of tea plants. Under different abiotic stresses, the two *CsF3H*s were induced remarkably by ultraviolet (UV) radiation, sucrose, and abscisic acid (ABA). In the seeds of *CsF3H*s transgenic *Arabidopsis thaliana*, the concentration of most flavonol glycosides and oligomeric proanthocyanidins increased significantly, while the content of monocatechin derivatives decreased. The present study revealed that *CsF3H*s played critical roles in flavonoid biosynthesis in tea plants.

## 1. Introduction

Flavonoids are widely distributed in plants, and have the medicinal functions of cerebral ischemia injury prevention, analgesia, and antineoplastic activity [1]. Therefore, they have been receiving more attention. In fresh tea leaves, 52 flavonoids have been isolated and identified [2], where catechins are the most abundant, accounting for 12–24% of the dry weight [3]. Flavonoids are synthesized through the phenylpropanoid pathway and the flavonoid biosynthesis pathway [4,5]. Flavanone 3-hydroxylase (F3H) (EC 1.14.11.9), which belongs to the 2-oxoglutarate-dependent dioxygenase (2-ODD) family, is the third enzyme of the central flavonoid biosynthetic pathway. F3H enzyme hydroxylates flavanones, such as naringenin, form 3-hydroxy flavonol, a common precursor of anthocyanins, flavanols, and proanthocyanidins [6,7]. F3H plays important roles in flavonoid biosynthesis [6,7]. In *Artemisia annua*, the F3H transcripts are found to be accumulated in the cultivar with higher level of flavonoids [8]. The high expression of *CtF3H* in quinochalcone-type safflower line is associated with the accumulation of flavonols [9]. Silencing *F3H* in *Malus sieversii* led to the accumulation of flavanone, the substrate of F3H [6]. In the seed coat, leaf, and stem of the *Arabidopsis thaliana F3H* mutant (*tt6*), the content of anthocyanin, the downstream product, was lower than that in the wild-type [10]. Furthermore, *F3H* also plays an important role in plant resistance to biotic and abiotic stresses [11,12]. Two *F3H* genes in *Reaumuria trigyna* were induced by drought, salt, cold, and abscisic acid (ABA) stresses [13]. Drought can induce *F3H* expression during potato growth [14], and *F3H* expression in Egyptian beans can provide resistance to the damage caused by fungus [15]. *F3H* in *Lycium chinense* played a role in drought tolerance, and its overexpression in tobacco enhanced tolerance to drought stress by improving the antioxidant system [16]. *SlF3HL* from *Solanum lycopersicum* conferred tolerance to chilling by stimulating flavonoid biosynthesis in transgenic tobacco [17]. *PnF3H* overexpression in *A. thaliana* improved tolerance to salt stress and ABA treatment by alleviating the inhibitory effects of naringenin on plant growth and changing the flavonoid components in transgenic plants [18]. In *Reaumuria soongorica*, the increases of *RsF3H* gene expression, RsF3H enzyme activity and the antioxidative ability of the metabolic end products in the flavonoid biosynthetic pathway enhanced the stress tolerance to ultraviolet (UV)-B radiation and drought [19].

The function of one *F3H* gene has been preliminarily studied in *Camellia sinensis* [20,21,22]; however, the total number of *F3H* members and their functions in flavonoid metabolism in tea plants remain unclear. According to current online databases of transcriptomes in the National Center for Biotechnology Information (NCBI) and genomes of the tea plant (www.plantkingdomgdb.com/tea_tree/) [23], two *CsF3H* transcripts were screened. In this study, a comprehensive analysis of these two *CsF3H*s was conducted including multiple sequence alignment, phylogenetic analysis, gene function verification, and response to abiotic stresses.

## 2. Materials and Methods

### 2.1. Plant Materials

Samples of *Camellia sinensis* cv. ‘Shuchazao’ at six different developmental stages (bud, first leaf, second leaf, third leaf, fourth leaf, and old leaf), young stem, and young root were collected from the agricultural research base of Anhui Agricultural University (Hefei, Anhui, China), immediately frozen in liquid nitrogen, then stored at −80 °C.

For the UV treatment, the approximately 12 cm tender shoots were illuminated under UV-B (0.6 W cm^−2^) for 30 and 60 min. The control shoots were illuminated under white light. 

With the abiotic stresses, shoots were treated under 90 mM/L sucrose, 200 mM/L mannitol, 50 mM/L NaCl, 10 μM/L jasmonic acid (JA), 0.74 μM/L indolebutyric acid (IBA), 100 μM/L ABA, and 20 mM/L salicylic acid (SA) for 12 and 48 h, respectively. All samples were treated at 22 °C with 150–200 μmol m^−2^ s^−1^ illumination intensity. The control shoots were cultivated in deionized water. For cold treatment, the shoots were treated at 4 °C for 3 and 6 h. 

For genetic transformation, *A. thaliana* (Ecotype Columbia) were grown in a green house. The temperature, light intensity and photoperiod were 22 ± 2 °C, 50 μmol m^−2^ s^−1^ and 16/8 h (light/darkness), respectively.

### 2.2. Clone, Multiple Alignment, and Phylogenetic Analysis

Two CsF3H transcripts were screened out after removing redundancies from nine transcriptome databases in NCBI and one genome database (www.plantkingdomgdb.com/tea_tree/) [23]. The fragments of 5′- and 3′-rapid amplification of cDNA ends (RACE) were cloned with primers shown in Supplementary Table S1 using the SMART RACE cDNA amplification kit (Clontech, Mountain View, CA, USA). The open reading frames (ORF) of these two genes were obtained by end-to-end PCR using specific primers (Supplementary Table S2). These two genes were named *CsF3Ha* and *CsF3Hb*, and submitted to GenBank (accession numbers: KY615688 and KY615689). 

Their theoretical molecular weight and isoelectric points were predicted using the ExPASy ProtParam tool [24]. By using ClustalW software (Kyoto University Bioinformatics Center, Kyoto, Japan) and the online server ESpript [25], an alignment of these two CsF3H proteins and F3H proteins from other species was implemented. Furthermore, a phylogenetic tree including these two CsF3H proteins and 2-ODD family members from other plants was constructed using MEGA 5.0 software (Mega, Raynham, MA, USA) through a neighbor-joining method.

### 2.3. Enzymatic Activity Assay of Recombinant CsF3H Proteins

The ORFs of *CsF3Ha* and *CsF3Hb* were respectively cloned into the expression vector pMAL-c2X (New England Biolabs, Ipswich, MA, USA) and transformed into *E. coli* BL21 expression strains (TransGen Biotech, Beijing, China). The expression strains grown in Luria–Bertani medium at 37 °C until the optical density (OD)_600_ reached 0.6 and 1.0 mM isopropyl β-d-1-thiogalactopyranoside (IPTG) was added to the medium and incubated for 5 h. The substrates, naringenin, and eriodictyol were then respectively added into the culture to a final concentration of 0.1 mM and incubated at 28 °C overnight. *E. coli* BL21 expression strains harboring the empty vector pMAL-c2X was used as a control. The culture was collected and centrifuged at 16,200× *g* for 10 min. The supernatant was filtered with a 0.22 μm membrane and analyzed by high-performance liquid chromatography (HPLC) at 280 nm. The chromatographic column was an Altima C18 analytical column (5 μm, 250 mm × 4.6 mm) (Agilent, Santa Clara, CA, USA). The moving phase was 1% acetic acid (A phase) and 100% acetonitrile (B phase). The flow velocity was 1.0 mL/min. The following linear elution gradient was employed: 0–5 min, B phase raised from 10 to 15%; 5–15 min, B phase raised from 15 to 40%; 15–20 min, B phase raised from 40 to 60%; and 20–22 min, B phase reduced from 60 to 10%. In addition, the ultra-performance liquid chromatography-tandem mass spectrometry (UPLC–MS/MS) system was utilized to identify the enzymatic products as previously described [2].

### 2.4. Expression Profile Analysis of CsF3Hs in Diverse Tissues and under Different Abiotic Stresses

To analyze the expression profiles of two *CsF3H*s in diverse tissues of the tea plant, we performed semi-quantitative reverse transcription-polymerase chain reaction (RT-PCR) for the first leaf, second leaf, third leaf, fourth leaf, old leaf, stem, and root with glyceraldehyde-3-phosphate dehydrogenase (*GAPDH*) as the internal reference gene.

In addition, real-time quantitative polymerase chain reaction (qPCR) was performed to investigate the expression profiles of two *CsF3H*s under abiotic stresses such as UV, ABA, SA, JA, IBA, NaCl, cold, sucrose, and mannitol. The second leaves were collected after treatment and stored at −80 °C. For qPCR, total RNA was extracted from the stored samples, and reverse transcription was performed using reverse transcriptase (TaKaRa). The gene-specific primers (Supplementary Table S3) were designed. Then, the cDNA was used as a template to perform qPCR on a Bio-Rad CFX96 Real-Time system (Bio-Rad, Hercules, CA, USA). *GAPDH* was used as the internal reference gene. The relative expression level of each gene was calculated using the 2^−ΔΔCT^ method [26].

For prediction of the *cis* elements, 2000 bp promoter regions of the two *CsF3H*s genes were analyzed using PlantCARE [27].

### 2.5. Analysis of Flavonoids in Transgenic Arabidopsis thaliana

The ORFs of *CsF3Ha* and *CsF3Hb* were respectively cloned into a binary vector using the Gateway Cloning System (Invitrogen, Carlsbad, CA, USA) [28]. The constructed binary expression vectors pCB2004-CsF3Ha and pCB2004-CsF3Hb were respectively introduced into the *Agrobacterium tumefaciens* strain C58C1 by electroporation. *A. thaliana* transformation was performed using the floral-dip method [29,30].

The weight of 0.1 g seeds of transgenic *A. thaliana* was ground into powder in liquid nitrogen. Flavonoids were extracted with 1 mL of methanol–HCl solution (80% methanol, 0.1% hydrochloric acid, and pure water). The extractive was intensively mixed with a methanol–HCl solution by ultrasonication. Finally, the mixture was centrifuged at 4000× *g* for 15 min, and the supernatant was moved into a clean microcentrifuge tube. The residues were re-extracted twice as above.

UPLC–MS/MS was used to analyze the flavonoids in the transgenic lines of *CsF3Ha* and *CsF3Hb*. The wild-type was used as a control. The methods of flavonoid identification and data analysis were derived from a previous study [2].

## 3. Results

### 3.1. Characterization of Two CsF3Hs

**Figure 1:**
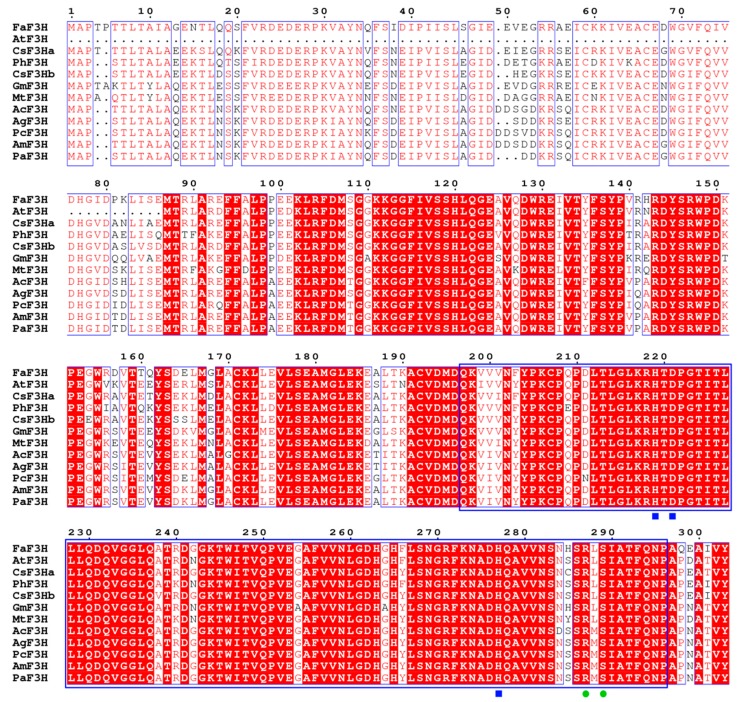

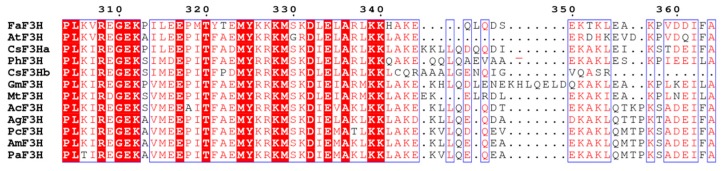
Multiple alignment of two CsF3H proteins with F3Hs from other plants. The blue and green symbols represent the iron binding sites and 2-oxoglutarate binding sites, respectively. The box represents the conserved area of the 2-oxoglutarate-dependent dioxygenase (2-ODD) family.

Two CsF3H transcripts (CsF3Ha and CsF3Hb) were screened out from the nine transcriptome databases and one genome database after removing redundancies. These two cDNAs of *CsF3H*s were obtained from *C. sinensis*, with lengths of 1256 and 1334 bp and open reading frames encoding 369 and 357 amino acids, respectively. The predicted protein molecular weight (Mw) and isoelectric point (pI) values are listed in Table 1. Multiple alignment analysis was further performed with these two CsF3H proteins and F3Hs from other plants (Figure 1). The result indicated that amino acid sequences of F3Hs from different plants were highly conservative. Like F3Hs of other plants, these two CsF3H proteins contained the 2-ODD conserved domain, which is specific to 2-ODD superfamily (Figure 1). The ferrous iron binding sites HxDxnH (H_218_, D_220_, and H_276_) and 2-oxoglutarate binding sites RxS (R_286_ and S_288_) were highly conserved in the F3Hs of *C. sinensis* and other plants (Figure 1).

**Figure 2:**
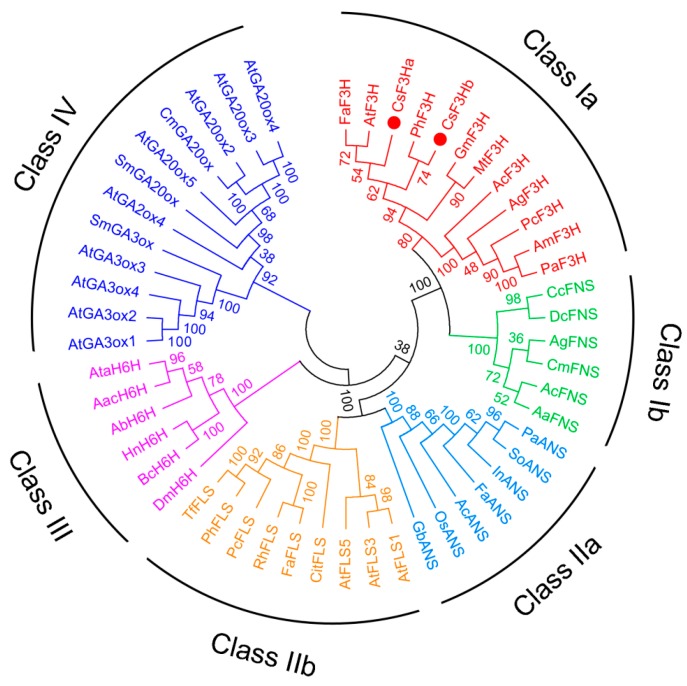
Phylogenetic relationship of CsF3Hs with 2-ODD family members from other plants. CsF3Hs are indicated by points (●). The following sequences were analyzed: FaF3H (*Fragaria ananassa*, AY691918.1), AtF3H (*Arabidopsis thaliana*, At3g51240), PhF3H (*Petunia hybrida*, AF022142), GmF3H (*Glycine max flavanone*, AY595420.1), MtF3H (*Medicago truncatula*, FJ529406.1), AcF3H (*Aethusa cynapium*, DQ683351.1), AgF3H (*Anethum graveolens*, AY817679.1), PcF3H (*Petroselinum crispum*, AY230248.1), AmF3H (*Ammi majus*, AY817678.1), PaF3H (*Pimpinella anisum*, AY817674.1), CcFNS (*Cuminum cyminum*, DQ683349.1), DcFNS (*Daucus carota*, AY817675.1), AgFNS (*Apium graveolens*, AY817676.1), CmFNS (*Conium maculatum*, AY817677.1), AcFNS (*Aethusa cynapium*, DQ683350.1), AaFNS (*Angelica archangelica*, DQ683352), PaANS (*Phytolacca americana*, AB198870.1), SoANS (*Spinacia oleracea*, AB198869.1), FaANS (*Fragaria x ananassa*, AY695817.1), InANS (*Ipomoea nil*, AB073925.1), AcANS (*Allium cepa*, EF192598.1), OsANS(*Oryza sativa*, Y07955.1), GbANS (*Ginkgo biloba*, ACC66092.1), AtFLS1 (*A. thaliana*, *At5g08640*), AtFLS3 (*A. thaliana*, *At5g63590*), AtFLS5 (*A. thaliana*, At5g63600), CitFLS (*Citrus unshiu*, AB011796), FaFLS (*Fragaria x ananassa*, DQ087252.1), RhFLS (*Rosa hybrida*, AB038247.1), PcFLS (*Petroselinum crispum*, AY230249.1), PhFLS (*Petunia hybrida*, Z22543.1), TfFLS (*Torenia fournieri*, AB078512.1), DmH6H (*Datura metel*, AF435417), BcH6H (*Brugmansia candida*, EU530633.1), HnH6H (*Hyoscyamus niger*, M62719), AbH6H (*Atropa baetica*, EF442802), AacH6H (*Anisodus acutangulus*, EF187826), AtaH6H (*Anisodus tanguticus*, AY356396.1), AtGA3ox1 (*A.thaliana*, At1g15550), AtGA3ox2 (*A.thaliana*, At1g80340), AtGA3ox4 (*A.thaliana*, At1g80330), AtGA3ox3 (*A.thaliana*, At4g21690), SmGA3ox (*Selaginella moellendorffii*, ABX10776.1), AtGA2ox4 (*A.thaliana*, At1g47990), SmGA20ox (*Selaginella moellendorffii*, ABX10768.1), AtGA20ox5 (*A.thaliana*, At1g44090), CmGA20ox (*Cucurbita maxima*, AAB64345), AtGA20ox2 (*A.thaliana*, At5g51810), AtGA20ox3 (*A.thaliana*, At5g07200), and AtGA20ox4 (*A. thaliana*, At1g60980).

A phylogenetic tree containing the two CsF3Hs and 2-ODD family members from other plants were constructed using MEGA 5.0 software (Figure 2). Phylogenetic analysis showed that these 2-ODD family members were classified into four clusters: classes I–IV. Class I contained flavanone 3-hydroxylase (Class Ia) and flavone synthase (Class Ib). Class II included anthocyanidin synthase (Class IIa) and flavonol synthase (Class IIb). Class III contained hyoscyamine 6β-hydroxylase. Class IV contained 20-oxidase and 3-oxidase. F3Hs and flavone synthases (FNSs) sharing the same cluster indicated that the F3Hs had a close evolutionary relationship with FNSs in plants (Figure 2). This result was consistent with previous reports that FNS evolved from F3H through gene duplication [31,32].

### 3.2. Enzymatic Activity Assay

**Figure 3:**
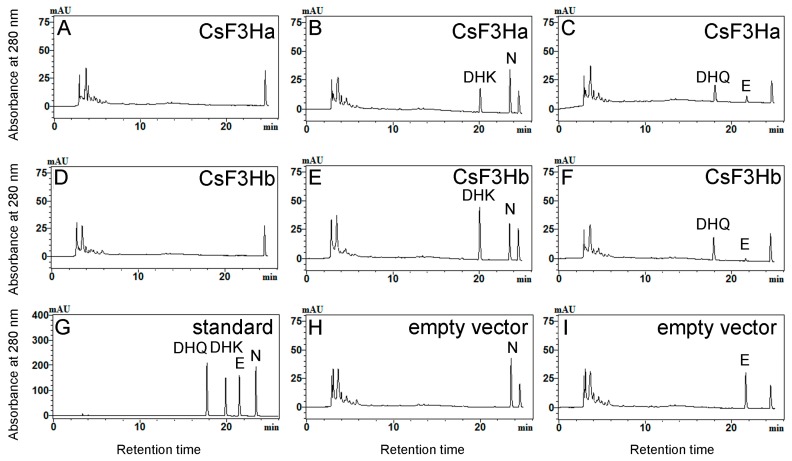
Enzymatic reaction products analysis of recombinant CsF3H proteins. (**A**) The control of (B) and (C) without substrate. (**B**,**C**) spectrums of reaction products from *Escherichia coli* strains harboring recombinant CsF3Ha-MBP with naringenin (N) and eriodictyol (E) as the substrate, respectively. (**D**) The control of (E) and (F) without substrate. (**E**,**F**) spectra of reaction products from *E. coli* strains harboring recombinant CsF3Hb-MBP with N and E as the substrate, respectively. (**G**) Mixed standard samples, including N, E, dihydrokaempferol (DHK), and dihydroquercetin (DHQ). (**H**,**I**) Control treatments with *E. coli* strains harboring empty vectors using N and E as substrate, respectively. mAU: 10^−3^ Absorbance unit.

**Figure 4:**
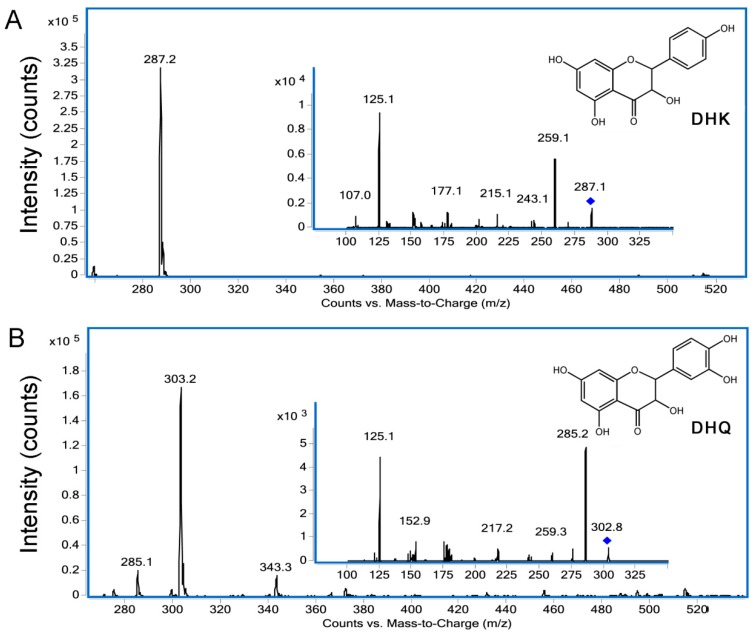
Identification of the enzymatic products by ultra-performance liquid chromatography-tandem mass spectrometry (UPLC–MS/MS) analysis. The products from *E. coli* strains harboring CsF3Ha/b in the pMAL-c2X vector with (**A**) N and (**B**) E as substrate, respectively. The diamond marks present precursor ions of DHK and DHQ respectively.

The CsF3Ha-maltose binding protein (MBP) and CsF3Hb-MBP recombinants were expressed in *E. coli* BL21 strains. No enzymatic activities were detected with purified proteins. Therefore, enzymatic activity assays were performed by feeding naringenin (N) and eriodictyol (E) as the substrate, respectively into the culture medium of the *E. coli* strains. The results showed that CsF3Ha and CsF3Hb were both able to catalyze N to form dihydrokaempferol (DHK) and E to form dihydroquercetin (DHQ) (Figure 3). Furthermore, UPLC–MS/MS analysis was used to identify the reaction products. The results confirmed that the products were DHK and DHQ, respectively (Figure 4).

### 3.3. Expression Profile Analysis

**Figure 5:**
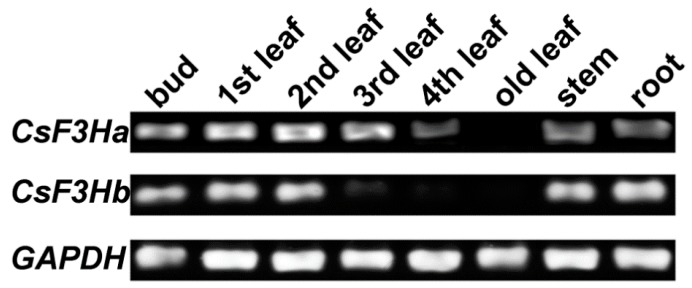
Semi-quantitative reverse transcription-polymerase chain reaction (RT-PCR) of *CsF3Hs* in diverse tissues of *Camellia sinensis*. Glyceraldehyde-3-phosphate dehydrogenase (*GAPDH*) was used as the internal reference gene. The experiments were performed with three biological repeats.

Semi-quantitative RT-PCR was used to analyze the expression profiles of *CsF3Ha* and *CsF3Hb* in various tissues. The results suggested that *CsF3Ha* and *CsF3Hb* exhibited high expression in the tender leaves such as the bud, first leaf, and second leaf, and low expression in the fourth and old leaves (Figure 5). Their expression profiles in leaves at different stages were consistent with flavonoid accumulation patterns in tea plants [2]. These results indicated that these two *CsF3Hs* most likely play important roles in flavonoid biosynthesis in tea plants.

**Figure 6:**
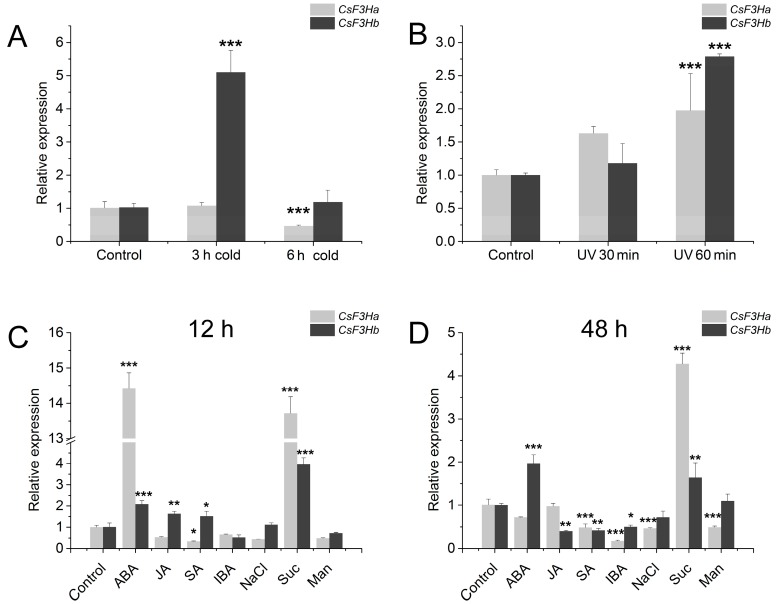
Relative expression patterns of two *CsF3Hs* under different abiotic stresses. (**A**) cold; (**B**) UV; (**C**,**D**) abscisic acid (ABA), jasmonic acid (JA), salicylic acid (SA), indolebutyric acid (IBA), NaCl, sucrose, and mannitol. All the data of real-time quantitative polymerase chain reaction (qPCR) were present based on three biological and technical repeats. Asterisk indicates significant difference compared with control samples (*n* = 3, * *p* < 0.05, ** *p* < 0.01, *** *p* < 0.001) based on a Tukey’s honestly significant difference test. Suc: Sucrose; Man: Mannitol.

The expression profiles of *CsF3Ha* and *CsF3Hb* under different abiotic stresses were detected using qPCR. The results showed that *CsF3Ha* and *CsF3Hb* were both significantly induced by UV at 60 min (Figure 6). Cs*F3Hb* was induced by cold treatment, while *CsF3Ha* was downregulated under cold treatment. *CsF3Ha* and *CsF3Hb* were also highly expressed under sucrose and ABA treatment. These results indicated that flavonoid biosynthesis may be induced by abiotic stresses, including sucrose, ABA and UV. In addition, the *cis* elements of the promoter regions of *CsF3H*a and *CsF3Hb* genes were analyzed (Supplementary Tables S4 and S5). Various light responsive elements were distributed in the promoter regions of both *CsF3Ha* and *CsF3Hb* genes, suggesting these two genes might be regulated by ultraviolet, which was consistent with our results. There were also some different *cis* elements between the promoter regions of *CsF3H*a and *CsF3Hb*, which may lead to very different expression under the same stress conditions. There were MYB binding sites in the promoter region of *CsF3Ha* (Supplementary Tables S4 and S5), but not in that of *CsF3Hb*, which implied that *CsF3Ha* may be regulated by the MYB transcription factor, while *CsF3Hb* was not [33,34,35].

### 3.4. Overexpression of CsF3Ha and CsF3Hb Improved the Accumulation of Flavonoids in Arabidopsis thaliana

**Figure 7:**
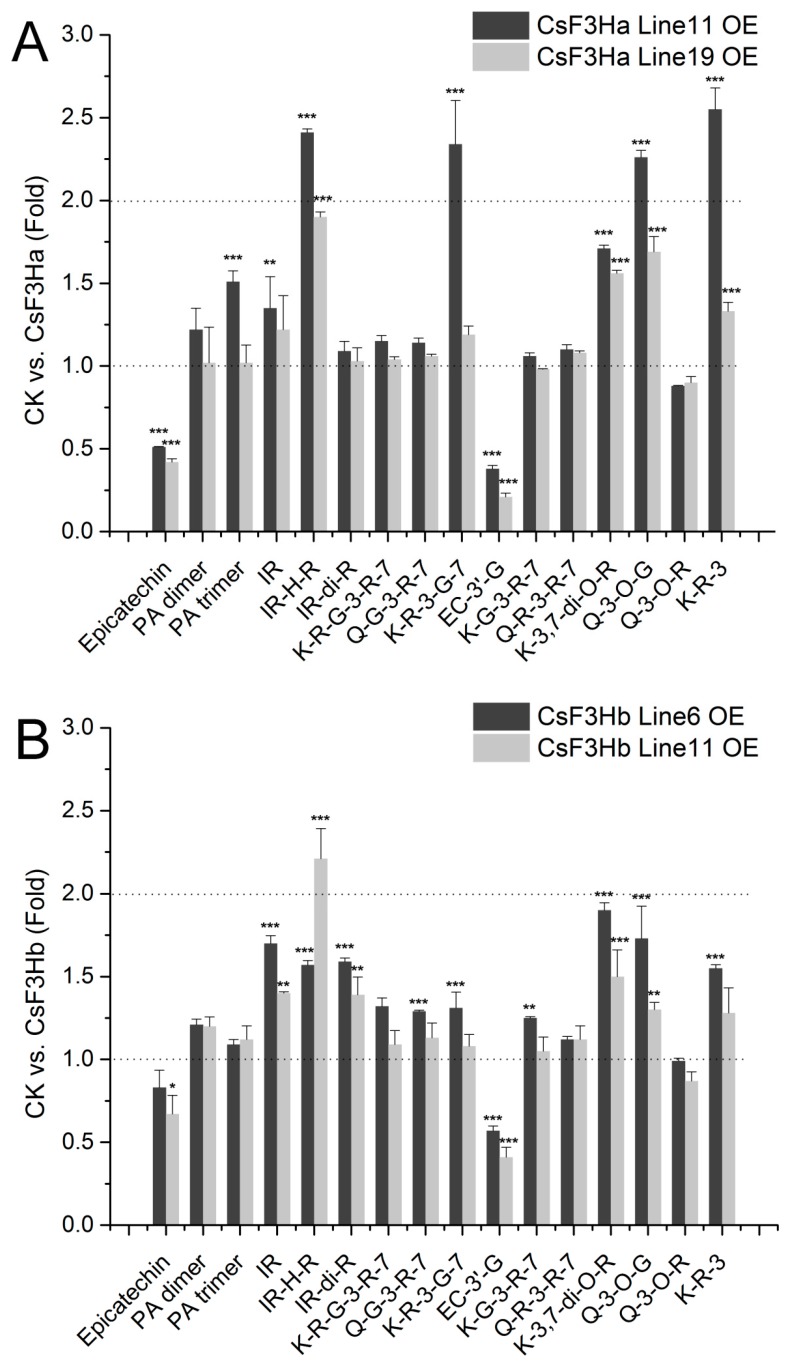
The accumulated flavonoid compounds in the seeds of transgenic (*CsF3Ha/b*) *A.thaliana*. Asterisk indicates significant difference compared with control samples (*n* = 3, * *p* < 0.05, ** *p* < 0.01, *** *p* < 0.001) based on a Tukey’s honestly significant difference test. G: Glucoside; H: Hexoside; I: Isorhamnetin; K: Kaempferol; Q: Quercetin; PA: Proanthocyanidin; R: Rhamnoside.

Flavonoids produced in transgenic *A. thaliana* lines were analyzed by UPLC–MS/MS. The results suggested that *CsF3Ha* and *CsF3Hb* overexpression significantly increased the production of most flavonol glycosides and oligomeric proanthocyanidins, such as Kaempferol 3-*O*-rhamnoside-7-*O*-glucoside (K-R-3-G-7), Kaempferol 3-*O*, 7-*O*-rhamnoside (K-3,7-di-O-R), quercetin 3-*O*-glucoside (Q-3-*O*-G), Kaempferol 3-*O*-rhamnoside (K-R-3), proanthocyanidin (PA) dimer, and PA trimer etc. (Figure 7 and Supplementary Table S6). Notably, flavonol glycosides K-R-3-G-7, Q-3-O-G, and K-R-3 in *CsF3Ha* Line11 OE were more than twice as much as that of the wild-type. However, the monocatechin derivatives, epicatechin and epicatechin 3'-*O*-glucoside (EC-3′-G) decreased in all the *CsF3Ha* and *CsF3Hb* transgenic *A. thaliana* lines. Therefore, *CsF3Ha* and *CsF3Hb* overexpression improved the accumulation of flavonoids in *A. thaliana*, except monocatechin derivatives.

## 4. Discussion

In flavonoid metabolic pathways, dihydroflavonols serve as intermediates for the biosynthesis of flavan-3-ols [36]. F3H, which converts flavanone into dihydroflavonol, is a key enzyme regulating plant flavonoid accumulation in vivo [37,38]. Numerous studies have reported that the F3H enzyme can catalyze naringenin to dihydrokaempferol [7,8,39], but the studies on its enzymatic activity for substrate E are rare. In this study, the enzymatic activity assay indicated that both the recombinant CsF3Ha and CsF3Hb in *E. coli* strains were able to catalyze N to form DHK, and E to form DHQ.

The two featured conserved motifs, namely ferrous iron binding sites HxDxnH (H_218_, D_220_, and H_276_) and 2-oxoglutarate binding sites RxS (R_286_ and S_288_), were highly conserved in these two CsF3Hs, which were consistent with the F3H protein from *A. annua* L. [8,40,41]. This indicated that these two CsF3H proteins belonged to the family of 2-ODD. Xiong et al. proposed that three strictly conserved prolines (Pro148, Pro204, and Pro207) played important roles in the protein-folding process [8]. The three prolines were also conserved in the two CsF3Hs, which may contribute to maintaining the functions of F3Hs during the evolutionary process of *C. sinensis*. In addition, phylogenetic analysis indicated that F3Hs had close evolutionary relationships with FNSs in plants, which was consistent with previous reports that *FNS* evolved from *F3H* by gene duplication [31,32]. Gebhardt et al. reported that several conserved differences between FNS and F3H proteins of parsley (*Petroselinum crispum*) were likely to determine divergent catalytic activity [42]. Both F3H and FNS withdrew the *β*-configured hydrogen from carbon-3 of naringenin, but then proceeded on different routes despite their high sequence similarity. F3H catalyzed 3β-hydroxylation through a rebound process, whereas FNS afforded the synelimination of hydrogen from carbon-2 in a cage-like setting without intermediate hydroxylation [42]. We examined the enzyme activity of CsF3Ha and CsF3Hb towards flavone, and the HPLC analysis showed that these two CsF3Hs did not exhibit enzyme activity of FNS (data not shown).

The expression of *F3H* gene contributes to the accumulation of downstream products, such as flavonols, catechins, PAs, and anthocyanins [6,7,9,10]. The transcript abundance of the flavonoid biosynthesis enzyme gene *F3H* was higher in the red petals than in the white petals of *Magnolia sprengeri* pamp [43]. In our work, many flavonoids including IR, IR-H-R, K-R-3-G-7, K-3,7-di-O-R, Q-3-O-G, and K-R-3 increased significantly in *CsF3Ha/b* transgenic *Arabidopsis thaliana*, which was consistent with previous report that *SlF3HL* overexpression induced flavonoid accumulation in tobacco [17]. This result may have been caused by the overexpression of *CsF3Ha*/*b*, which increased the metabolic flux toward flavonoid pathway. In transgenetic *SlF3HL* tobacco, *CHS*, and *CHI* in the upstream pathway and *FLS* in the downstream pathway were upregulated by *SlF3HL* overexpression [17]. 

Notably, oligomeric proanthocyanidins, such as PA dimer and PA trimer increased in *CsF3Ha/b* transgenic *A. thaliana*. However, the monocatechin types, epicatechin and EC-3’-G, were reduced in all the transgenic *A. thaliana* lines. These results were inconsistent with those of a previous report [20]. Monika Mahajan reported that the overexpression of *CsF3H*, equal to *CsF3Ha* in this study, increased the content of flavan-3-ols, including catechin, epicatechin (EC), and epigallocatechin (EGC) in tobacco [20]. These differences may come from the differences of the transgenic plants. The main types of flavan-3-ols present in the seeds of *Arabidopsis* are PAs (catechin polymer) instead of monocatechins. Epicatechin may polymerize to form PAs in transgenic *A. thaliana*. Due to the lack of an effective genetic transformation in tea plants, the functions of *F3H*s for the biosynthesis of PAs and monocatechin in tea plants remains unclear. However, the present study revealed that the two *CsF3H*s play critical roles in the flavonoid biosynthesis in tea plants.

Only one *F3H* gene was reported in most papers, while few papers reported more than two *F3H* genes, such as three *F3H* genes were found in rice and wheat. Their expression patterns in tissues may be different, implying functional difference. For instance, *OsF3H-1* and *-2* are expressed at much higher levels than *OsF3H-3* in rice tissues producing high contents of anthocyanins [44]. For all three wheat *F3H* genes, *F3H-A1*, *F3H-B1*, and *F3H-D1*, there was a close relationship between their expression and tissue pigmentation, and they were highly activated in the red grains and coleoptiles [45]. In this study, a comparison with *CsF3Ha* and *CsF3Hb* showed that the transcript level of *CsF3Ha* was higher in the third and fourth leaves, while that of *CsF3Hb* was slightly higher in stems and roots, suggesting that *CsF3Hb* may contribute to polymeric catechins [2]. 

In plants, sucrose is an essential metabolite, not only playing an essential role in general metabolism and energy generation, but also acting as a signaling molecule to regulate processes, such as photosynthesis, nutrient mobilization and allocation, stimulating growth, and activating the flavonoid biosynthesis (sugar sensing and signaling in plants.). As a signaling molecule, sugar induced the expressions of genes specifically involved in anthocyanin biosynthesis in *A. thaliana* seedlings, such as *PAL*, *C4H*, *4CL*, *CHS*, *CHI*, *F3H*, *F3’H*, *DFR*, *LAR*, *ANS*, *ANR*, *UFGT*, *FLS*, and *PAP1* [46,47]. The amount of anthocyanin in *Arabidopsis* seedlings reached its maximum under the treated sucrose concentration of 60–90 mM [46]. In our previous work, sucrose also induced an increase in catechins in tea plant seedlings [48]. In this work, *CsF3Ha* and *CsF3Hb* were significantly induced, especially *CsF3Ha* under the treatment of 90 mM sucrose, suggesting *CsF3Ha* may contribute more to activating the flavonoid biosynthesis. *F3H* also plays an important role in the plant resistance to some abiotic stresses, such as drought, salt, cold, ABA, and UV stresses [13,14,17,18,19]. Combining previous reports [17,35,49,50] and pretreatments, 10 μM/L JA, 0.74 μM/L IBA, 100 μM/L ABA, and 20 mM/L SA were used to examine the ability of the two *CsF3Hs* to abiotic stresses in this paper. The results suggested that *CsF3Ha* and *CsF3Hb* were more strongly induced by ABA at 12 h after treatment than at 24 h, which was consistent with a previous study of *RtF3H1* and *RtF3H2* [13]. *CsF3Ha* was more highly induced than *CsF3Hb* under ABA. *CsF3Ha* was downregulated by NaCl and mannitol, while *CsF3Hb* expression exhibited no significant change under NaCl and mannitol. *CsF3Hb* was highly induced by cold at 3 h, suggesting that *CsF3Hb* may be involved in resistance to cold stress. By contrast, *RtF3H1* and *RtF3H2* could be induced by NaCl, polyethylene glycol (PEG), and cold [13]. Compared with *CsF3Ha*, *CsF3Hb* was more highly upregulated by UV. These phenomena may be caused by more *cis*-acting regulatory elements involved in light responsiveness in the promoter regions of *CsF3Hb*. These data suggested that these two *CsF3H* genes are involved in the abiotic stress of tea plants, A previous report has suggested that the increase of *RsF3H* gene expression, RsF3H enzyme activity and the antioxidative ability of the metabolic end products in the flavonoid biosynthetic pathway led to the stress tolerance of *R. soongorica* [19].

## Figures and Tables

**Table 1:** The basic information of two *CsF3H* genes.

Gene Name	Accession Number	Mw (kD)	cDNA Length (bp)	ORF length (bp)	5′-UTR Length (bp)	3′-UTR Length (bp)	Size (aa)	pI
*CsF3Ha*	KY615688	41.46	1256	1107	54	95	369	5.61
*CsF3Hb*	KY615689	39.92	1334	1071	84	179	357	5.23

aa: amino acids; Mw: Molecular weight; cDNA: complementary DNA; UTR: untranslated region; ORF: Open reading frame; pI: Isoelectric point.

## Supplementary Materials

The following are available online at www.mdpi.com/2073-4425/8/11/300/s1. Table S1: The specific-primers for RACE; Table S2: The specific-primers for ORFs; Table S3: The specific-primers for qPCR; Table S4: The *cis* elements of promoter regions of *CsF3H*a; Table S5: The *cis* elements of promoter regions of *CsF3Hb*, Table S6: Levels of selected flavonoid compounds in seeds of wild-type and transgenic (*CsF3Ha/b*) *Arabidopsis thaliana* lines determined by UPLC-MS analysis.
