# Supplementary material for: Functional Analysis of Two Flavanone-3-Hydroxylase Genes from Camellia sinensis: A Critical Role in Flavonoid Accumulation
Source: Genes (Basel). 2017 Oct 31;8(11):300. doi: 10.3390/genes8110300 (PMC5704213; doi:10.3390/genes8110300)
Supplement: Supplementary file 1 [file genes-08-00300-s001.zip › Table S4.docx]

Table S4 The cis elements of promoter regions of *CsF3H*a

| **Site Name** | **Position** | **function** |  |  |  |  |  |  |
| --- | --- | --- | --- | --- | --- | --- | --- | --- |
| [3-AF1 binding site](http://bioinformatics.psb.ugent.be/webtools/plantcare/cgi-bin/show_site_info.htpl?QWhere=ID_of_Site%20like%20%27ST~3-AF1%20binding%20site%27&StartAt=0&NbRecs=10) | -813 | light responsive element |  |  |  |  |  |  |
| [AT1-motif](http://bioinformatics.psb.ugent.be/webtools/plantcare/cgi-bin/show_site_info.htpl?QWhere=ID_of_Site%20like%20%27ST~AT1-motif%27&StartAt=0&NbRecs=10) | -1142 | part of a light responsive module |  |  |  |  |  |  |
| [Box 4](http://bioinformatics.psb.ugent.be/webtools/plantcare/cgi-bin/show_site_info.htpl?QWhere=ID_of_Site%20like%20%27PC~Box%204%27&StartAt=0&NbRecs=10) | -1136 | part of a conserved DNA module involved in light responsiveness |  |  |  |  |  |  |
| [Box 4](http://bioinformatics.psb.ugent.be/webtools/plantcare/cgi-bin/show_site_info.htpl?QWhere=ID_of_Site%20like%20%27PC~Box%204%27&StartAt=0&NbRecs=10) | -669 | part of a conserved DNA module involved in light responsiveness |  |  |  |  |  |  |
| [Box I](http://bioinformatics.psb.ugent.be/webtools/plantcare/cgi-bin/show_site_info.htpl?QWhere=ID_of_Site%20like%20%27PS~Box%20I%27&StartAt=0&NbRecs=10) | -646 | light responsive element |  |  |  |  |  |  |
| [Box I](http://bioinformatics.psb.ugent.be/webtools/plantcare/cgi-bin/show_site_info.htpl?QWhere=ID_of_Site%20like%20%27PS~Box%20I%27&StartAt=0&NbRecs=10) | -522 | light responsive element |  |  |  |  |  |  |
| [G-Box](http://bioinformatics.psb.ugent.be/webtools/plantcare/cgi-bin/show_site_info.htpl?QWhere=ID_of_Site%20like%20%27PS~G-Box%27&StartAt=0&NbRecs=10) | -1454 | cis-acting regulatory element involved in light responsiveness |  |  |  |  |  |  |
| [GAG-motif](http://bioinformatics.psb.ugent.be/webtools/plantcare/cgi-bin/show_site_info.htpl?QWhere=ID_of_Site%20like%20%27ZM~G-box%27&StartAt=0&NbRecs=10) | -67 | part of a light responsive element |  |  |  |  |  |  |
| [GT1-motif](http://bioinformatics.psb.ugent.be/webtools/plantcare/cgi-bin/show_site_info.htpl?QWhere=ID_of_Site%20like%20%27AT~GT1-motif%27&StartAt=0&NbRecs=10) | -288 | light responsive element |  |  |  |  |  |  |
| [GT2-motif](http://bioinformatics.psb.ugent.be/webtools/plantcare/cgi-bin/show_site_info.htpl?QWhere=ID_of_Site%20like%20%27AT~GT1-motif%27&StartAt=0&NbRecs=10) | -261 | light responsive element |  |  |  |  |  |  |
| [I-box](http://bioinformatics.psb.ugent.be/webtools/plantcare/cgi-bin/show_site_info.htpl?QWhere=ID_of_Site%20like%20%27ST~I-box%27&StartAt=0&NbRecs=10) | -564 | part of a light responsive element |  |  |  |  |  |  |
| [Sp1](http://bioinformatics.psb.ugent.be/webtools/plantcare/cgi-bin/show_site_info.htpl?QWhere=ID_of_Site%20like%20%27ZM~Sp1%27&StartAt=0&NbRecs=10) | -223 | light responsive element |  |  |  |  |  |  |
| [TCCC-motif](http://bioinformatics.psb.ugent.be/webtools/plantcare/cgi-bin/show_site_info.htpl?QWhere=ID_of_Site%20like%20%27SO~TCCC-motif%27&StartAt=0&NbRecs=10) | -38 | part of a light responsive element |  |  |  |  |  |  |
| TCT-motif | -692 | part of a light responsive element |  |  |  |  |  |  |
|  |  |  |  |  |  |  |  |  |
| [5UTR Py-rich stretch](http://bioinformatics.psb.ugent.be/webtools/plantcare/cgi-bin/show_site_info.htpl?QWhere=ID_of_Site%20like%20%27LE~5UTR%20Py-rich%20stretch%27&StartAt=0&NbRecs=10) | -789 | cis-acting element conferring high transcription levels |  |  |  |  |  |  |
| [5UTR Py-rich stretch](http://bioinformatics.psb.ugent.be/webtools/plantcare/cgi-bin/show_site_info.htpl?QWhere=ID_of_Site%20like%20%27LE~5UTR%20Py-rich%20stretch%27&StartAt=0&NbRecs=10) | -24 | cis-acting element conferring high transcription levels |  |  |  |  |  |  |
| [5UTR Py-rich stretch](http://bioinformatics.psb.ugent.be/webtools/plantcare/cgi-bin/show_site_info.htpl?QWhere=ID_of_Site%20like%20%27LE~5UTR%20Py-rich%20stretch%27&StartAt=0&NbRecs=10) | -28 | cis-acting element conferring high transcription levels |  |  |  |  |  |  |
| [5UTR Py-rich stretch](http://bioinformatics.psb.ugent.be/webtools/plantcare/cgi-bin/show_site_info.htpl?QWhere=ID_of_Site%20like%20%27LE~5UTR%20Py-rich%20stretch%27&StartAt=0&NbRecs=10) | -20 | cis-acting element conferring high transcription levels |  |  |  |  |  |  |
| [5UTR Py-rich stretch](http://bioinformatics.psb.ugent.be/webtools/plantcare/cgi-bin/show_site_info.htpl?QWhere=ID_of_Site%20like%20%27LE~5UTR%20Py-rich%20stretch%27&StartAt=0&NbRecs=10) | -786 | cis-acting element conferring high transcription levels |  |  |  |  |  |  |
| [5UTR Py-rich stretch](http://bioinformatics.psb.ugent.be/webtools/plantcare/cgi-bin/show_site_info.htpl?QWhere=ID_of_Site%20like%20%27LE~5UTR%20Py-rich%20stretch%27&StartAt=0&NbRecs=10) | -22 | cis-acting element conferring high transcription levels |  |  |  |  |  |  |
| [5UTR Py-rich stretch](http://bioinformatics.psb.ugent.be/webtools/plantcare/cgi-bin/show_site_info.htpl?QWhere=ID_of_Site%20like%20%27LE~5UTR%20Py-rich%20stretch%27&StartAt=0&NbRecs=10) | -26 | cis-acting element conferring high transcription levels |  |  |  |  |  |  |
| [Skn-1_motif](http://bioinformatics.psb.ugent.be/webtools/plantcare/cgi-bin/show_site_info.htpl?QWhere=ID_of_Site%20like%20%27OS~Skn-1_motif%27&StartAt=0&NbRecs=10) | -856 | cis-acting regulatory element required for endosperm expression |  |  |  |  |  |  |
| [GCN4_motif](http://bioinformatics.psb.ugent.be/webtools/plantcare/cgi-bin/show_site_info.htpl?QWhere=ID_of_Site%20like%20%27OS~GCN4_motif%27&StartAt=0&NbRecs=10) | -1232 | cis-regulatory element involved in endosperm expression |  |  |  |  |  |  |
|  |  |  |  |  |  |  |  |  |
| [MRE](http://bioinformatics.psb.ugent.be/webtools/plantcare/cgi-bin/show_site_info.htpl?QWhere=ID_of_Site%20like%20%27PC~MRE%27&StartAt=0&NbRecs=10) | -1057 | MYB binding site involved in light responsiveness |  |  |  |  |  |  |
| [MRE](http://bioinformatics.psb.ugent.be/webtools/plantcare/cgi-bin/show_site_info.htpl?QWhere=ID_of_Site%20like%20%27PC~MRE%27&StartAt=0&NbRecs=10) | -259 | MYB binding site involved in light responsiveness |  |  |  |  |  |  |
| [MBS](http://bioinformatics.psb.ugent.be/webtools/plantcare/cgi-bin/show_site_info.htpl?QWhere=ID_of_Site%20like%20%27AT~MBS%27&StartAt=0&NbRecs=10) | -56 | MYB binding site involved in drought-inducibility |  |  |  |  |  |  |
|  |  |  |  |  |  |  |  |  |
| [HSE](http://bioinformatics.psb.ugent.be/webtools/plantcare/cgi-bin/show_site_info.htpl?QWhere=ID_of_Site%20like%20%27BO~HSE%27&StartAt=0&NbRecs=10) | -1082 | cis-acting element involved in heat stress responsiveness |  |  |  |  |  |  |
| [LTR](http://bioinformatics.psb.ugent.be/webtools/plantcare/cgi-bin/show_site_info.htpl?QWhere=ID_of_Site%20like%20%27HV~LTR%27&StartAt=0&NbRecs=10) | -1180 | cis-acting element involved in low-temperature responsiveness |  |  |  |  |  |  |
| [ARE](http://bioinformatics.psb.ugent.be/webtools/plantcare/cgi-bin/show_site_info.htpl?QWhere=ID_of_Site%20like%20%27ZM~ARE%27&StartAt=0&NbRecs=10) | -1045 | cis-acting regulatory element essential for the anaerobic induction |  |  |  |  |  |  |
|  |  |  |  |  |  |  |  |  |
| [circadian](http://bioinformatics.psb.ugent.be/webtools/plantcare/cgi-bin/show_site_info.htpl?QWhere=ID_of_Site%20like%20%27LE~circadian%27&StartAt=0&NbRecs=10) | -625 | cis-acting regulatory element involved in circadian control |  |  |  |  |  |  |
| [circadian](http://bioinformatics.psb.ugent.be/webtools/plantcare/cgi-bin/show_site_info.htpl?QWhere=ID_of_Site%20like%20%27LE~circadian%27&StartAt=0&NbRecs=10) | -375 | cis-acting regulatory element involved in circadian control |  |  |  |  |  |  |
|  |  |  |  |  |  |  |  |  |
| [TCA-element](http://bioinformatics.psb.ugent.be/webtools/plantcare/cgi-bin/show_site_info.htpl?QWhere=ID_of_Site%20like%20%27BO~TCA-element%27&StartAt=0&NbRecs=10) | -765 | cis-acting element involved in salicylic acid responsiveness |  |  |  |  |  |  |
