# Supplementary material for: Functional Analysis of Two Flavanone-3-Hydroxylase Genes from Camellia sinensis: A Critical Role in Flavonoid Accumulation
Source: Genes (Basel). 2017 Oct 31;8(11):300. doi: 10.3390/genes8110300 (PMC5704213; doi:10.3390/genes8110300)
Supplement: Supplementary file 1 [file genes-08-00300-s001.zip › Table S5.docx]

Table S5 The cis elements of promoter regions of *CsF3Hb*

| **Site Name** | **Position** | **function** |  |  |
| --- | --- | --- | --- | --- |
| ABRE | -951 | cis-acting element involved in the abscisic acid responsiveness |  |  |
| ABRE | -175 | cis-acting element involved in the abscisic acid responsiveness |  |  |
| ABRE | -377 | cis-acting element involved in the abscisic acid responsiveness |  |  |
| ABRE | -949 | cis-acting element involved in the abscisic acid responsiveness |  |  |
| ABRE | -361 | cis-acting element involved in the abscisic acid responsiveness |  |  |
| motif IIb | -153 | abscisic acid responsive element |  |  |
| CGTCA-motif | -815 | cis-acting regulatory element involved in the MeJA-responsiveness |  |  |
| TGACG-motif | -815 | cis-acting regulatory element involved in the MeJA-responsiveness |  |  |
| GARE-motif | -233 | gibberellin-responsive element |  |  |
| GARE-motif | -47 | gibberellin-responsive element |  |  |
| TATC-box | -1005 | cis-acting element involved in gibberellin-responsiveness |  |  |
| TGA-element | -1222 | auxin-responsive element |  |  |
|  |  |  |  |  |
| ATCT-motif | -532 | part of a conserved DNA module involved in light responsiveness |  |  |
| Box 4 | -1241 | part of a conserved DNA module involved in light responsiveness |  |  |
| Box 4 | -829 | part of a conserved DNA module involved in light responsiveness |  |  |
| G-Box | -949 | cis-acting regulatory element involved in light responsiveness |  |  |
| G-Box | -377 | cis-acting regulatory element involved in light responsiveness |  |  |
| G-box | -949 | cis-acting regulatory element involved in light responsiveness |  |  |
| G-box | -377 | cis-acting regulatory element involved in light responsiveness |  |  |
| I-box | -299 | part of a light responsive element |  |  |
| I-box | -113 | part of a light responsive element |  |  |
| Sp1 | -217 | light responsive element |  |  |
| Sp1 | -31 | light responsive element |  |  |
| Sp1 | -184 | light responsive element |  |  |
| GT1-motif | -1253 | light responsive element |  |  |
|  |  |  |  |  |
| HSE | -1061 | cis-acting element involved in heat stress responsiveness |  |  |
| HSE | -741 | cis-acting element involved in heat stress responsiveness |  |  |
| TC-rich repeats | -1039 | cis-acting element involved in defense and stress responsiveness |  |  |
|  |  |  |  |  |
| AACA_motif | -263 | involved in endosperm-specific negative expression |  |  |
| AACA_motif | -77 | involved in endosperm-specific negative expression |  |  |
|  |  |  |  |  |
| circadian | -1135 | cis-acting regulatory element involved in circadian control |  |  |
