# Supplementary material for: Functional Analysis of Two Flavanone-3-Hydroxylase Genes from Camellia sinensis: A Critical Role in Flavonoid Accumulation
Source: Genes (Basel). 2017 Oct 31;8(11):300. doi: 10.3390/genes8110300 (PMC5704213; doi:10.3390/genes8110300)
Supplement: Supplementary file 1 [file genes-08-00300-s001.zip › Table S6.docx]

**Table S6** **Levels of selected flavonoid compounds in seeds of wild-type and transgenic (*CsF3Ha/b*) *Arabidopsis thaliana* lines determined by UPLC-MS analysis**

The datas represent peak areas.

|  | WT | F3HaLine11 | Ratio F3Haline11/ WT | F3HaLine19 | Ratio F3Haline19/ WT | F3HbLine6 | Ratio F3Hbline6/ WT | F3HbLine11 | Ratio F3Hbline11/ WT |
| --- | --- | --- | --- | --- | --- | --- | --- | --- | --- |
| Epicatechin | 363.5 | 184.67 | 0.51 | 151 | 0.42 | 303.33 | 0.83 | 242 | 0.67 |
| PA dimer | 1902 | 2327.67 | 1.22 | 1940 | 1.02 | 2296.33 | 1.21 | 2286 | 1.2 |
| PA trimer | 2280.5 | 3432.33 | 1.51 | 2317.33 | 1.02 | 2482.67 | 1.09 | 2547.33 | 1.12 |
|  |  |  |  |  |  |  |  |  |  |
| IR | 323.5 | 438 | 1.35 | 395 | 1.22 | 549.67 | 1.7 | 453.67 | 1.4 |
| IR-H-R | 6639.5 | 15984 | 2.41 | 12624 | 1.9 | 10432.67 | 1.57 | 14699.3 | 2.21 |
| IR-di-R | 21895.5 | 23882.33 | 1.09 | 22530.33 | 1.03 | 34724.67 | 1.59 | 30481.7 | 1.39 |
|  |  |  |  |  |  |  |  |  |  |
| K-R-G-3-R-7 | 354 | 405.67 | 1.15 | 369.67 | 1.04 | 466 | 1.32 | 387.33 | 1.09 |
| Q-G-3-R-7 | 198616 | 225658 | 1.14 | 210932.3 | 1.06 | 255628.5 | 1.29 | 224379 | 1.13 |
| K-R-3-G-7 | 172 | 402.67 | 2.34 | 205.33 | 1.19 | 226 | 1.31 | 186.33 | 1.08 |
| EC-3'-G | 5304 | 1989 | 0.38 | 1108.32 | 0.21 | 3010 | 0.57 | 2179 | 0.41 |
| K-G-3-R-7 | 19342 | 20423.33 | 1.06 | 18869 | 0.98 | 24138.33 | 1.25 | 20297 | 1.05 |
| Q-R-3-R-7 | 367836 | 406134.67 | 1.1 | 396842 | 1.08 | 410155 | 1.12 | 413257 | 1.12 |
| K-3,7-di-O-R | 37299 | 63633.33 | 1.71 | 58284.67 | 1.56 | 70969.67 | 1.9 | 55965.7 | 1.5 |
| Q-3-O-G | 1914.5 | 4327 | 2.26 | 3228.67 | 1.69 | 3320 | 1.73 | 2493 | 1.3 |
| Q-3-O-R | 523717 | 462610.33 | 0.88 | 469335 | 0.9 | 518248.3 | 0.99 | 455167 | 0.87 |
| K-R-3 | 1228 | 3132 | 2.55 | 1639.33 | 1.33 | 1903.67 | 1.55 | 1575 | 1.28 |

G, glucoside; H, hexoside; I, isorhamnetin; K, kaempferol; Q, quercetin; PA, proanthocyanidin; R, rhamnoside.
