# Supplementary material for: Functional Analysis of Two Flavanone-3-Hydroxylase Genes from Camellia sinensis: A Critical Role in Flavonoid Accumulation
Source: Genes (Basel). 2017 Oct 31;8(11):300. doi: 10.3390/genes8110300 (PMC5704213; doi:10.3390/genes8110300)
Supplement: Supplementary file 1 [file genes-08-00300-s001.zip › Table S1.doc]

Table S1 The specific-primers for RACE

| **Gene name** | **Primer 5´-->3´** |
| --- | --- |
| CsF3Ha 5’race inner | TCTTCTGCGAGAGCCGTAAGCGTTG |
| CsF3Ha 5’race outer | TGAGATCACCGGAATTTCATTGCTG |
| CsF3Ha 3’race inner | AGAGAAGAAGCTATTGCAAGATCAG |
| CsF3Ha 3’race outer | TTGAAGAGCCAATCACGTTCGCGGA |
| CsF3Hb 5’inner | GCGACCTTGGGACGCTCGTCCTCGT |
| CsF3Hb 5’outer | TCTTCTTACAGATCTCCGATCTCTT |
| CsF3Hb 3’inner | GGAAGATGAGCAAAGACCTTGAACT |
| CsF3Hb 3’outer | CAGATTATCCATAGCCACATTCCAG |
