# Supplementary material for: Functional Analysis of Two Flavanone-3-Hydroxylase Genes from Camellia sinensis: A Critical Role in Flavonoid Accumulation
Source: Genes (Basel). 2017 Oct 31;8(11):300. doi: 10.3390/genes8110300 (PMC5704213; doi:10.3390/genes8110300)
Supplement: Supplementary file 1 [file genes-08-00300-s001.zip › Table S2.doc]

Table S2 The specific-primers for ORFs

| **Gene name** | **Primer 5´-->3´** |
| --- | --- |
| CsF3HaF | ATGGCGCCAACAACAACGC |
| CsF3HaR | AGCAAAAATCTCATCAGTGC |
| CsF3HbF | ATGGCACCGTCCACACTGAC |
| CsF3HbR | TCTACTGGCTTGGACTCCAATTTG |
